# Tumor-Associated Macrophages in Glioma: Friend or Foe?

**DOI:** 10.1155/2013/486912

**Published:** 2013-05-08

**Authors:** Benjamin C. Kennedy, Christopher R. Showers, David E. Anderson, Lisa Anderson, Peter Canoll, Jeffrey N. Bruce, Richard C. E. Anderson

**Affiliations:** ^1^The Gabriele Bartoli Brain Tumor Research Laboratory, Department of Neurological Surgery, The Neurological Institute, Columbia University College of Physicians and Surgeons, New York City, NY 10032, USA; ^2^Erinyes Biotechnologies LLC, Boston, MA 02118, USA

## Abstract

Tumor-associated macrophages (TAMs) contribute substantially to the tumor mass of gliomas and have been shown to play a major role in the creation of a tumor microenvironment that promotes tumor progression. Shortcomings of attempts at antiglioma immunotherapy may result from a failure to adequately address these effects. Emerging evidence supports an independent categorization of glioma TAMs as alternatively activated M2-type macrophages, in contrast to classically activated proinflammatory M1-type macrophages. These M2-type macrophages exert glioma-supportive effects through reduced anti-tumor functions, increased expression of immunosuppressive mediators, and nonimmune tumor promotion through expression of trophic and invasion-facilitating substances. Much of our work has demonstrated these features of glioma TAMs, and together with the supporting literature will be reviewed here. Additionally, the dynamics of glioma cell-TAM interaction over the course of tumor development remain poorly understood; our efforts to elucidate glioma cell-TAM dynamics are summarized. Finally, the molecular pathways which underlie M2-type TAM polarization and gene expression similarly require further investigation, and may present the most potent targets for immunotherapeutic intervention. Highlighting recent evidence implicating the transcription factor STAT3 in immunosuppressive tumorigenic glioma TAMs, we advocate for gene array-based approaches to identify yet unappreciated expression regulators and effector molecules important to M2-type glioma TAMs polarization and function within the glioma tumor microenvironment.

## 1. Introduction 

Malignant glioma is uniformly fatal with a median survival of less than 15 months with aggressive treatment [1]. Advances in surgical, radiation, and conventional chemotherapeutic therapies have had minimal impact on the prognosis of this aggressive disease [1]. The recalcitrance of malignant glioma to standard therapies is believed to result from phenotypic heterogeneity and diffuse infiltration into normal brain parenchyma [2], as well as residence within the unique immune environment of the central nervous system (CNS) [3]. Long viewed as an “immune-privileged” site due to a perceived lack of specialized antigen presenting cells (APCs), restriction from circulating lymphocytes and other immune mediators by the blood brain barrier (BBB), and absence of lymphatic drainage [4], the CNS appeared to possess little immunologic potential to resist glioma progression. Evidence accumulated over the last 20 years, however, has largely debunked this view of the CNS by demonstrating distinct immune activation cascades within the CNS in response to cerebral ischemia and traumatic brain injury [5, 6], contingent upon activation of resident microglia and infiltrating macrophages capable of effective antigen presentation and lymphocyte activation [6–8], all permissible through inducible permeability of the BBB to leukocytes and immune mediators in pathological states [9]. Activated microglia have been shown to express phenotypic and functional characteristics of both macrophages and dendritic cells [10], and furthermore are capable of inducing T-cell responses in a mixed lymphocyte-type reaction *in vitro* [11]. Additionally, circulating tumor-specific antibodies and cytotoxic T lymphocytes (CTL) have been isolated from the peripheral blood of patients with malignant glioma [12], indicating the potential for a competent tumoricidal immune response to glioma within the CNS. This expanding appreciation of intrinsic CNS immune capacity against glioma, coupled with the limited efficacy and profound side effects of current glioma therapies, has prompted a major investigation into immunotherapy as a therapeutic strategy against glioma. 

The immune response to tumor-associated antigens and mechanisms of immunosuppression by tumor cells have been the most actively investigated areas of cancer immunotherapy research. These efforts have led to FDA-approved immunotherapy-based treatment protocols that have been shown to reduce tumor burden and prolong survival in patients with many different systemic tumors [13] including advanced prostate cancer and late stage melanoma [14, 15]. Unfortunately, immunotherapy against malignant glioma has so far met with only limited success [16]. At this time, the majority of immunotherapeutic efforts against malignant gliomas have focused on methods to try to stimulate an effective adaptive T-cell response against glioma tumor antigens. This is largely because there is ample evidence demonstrating lymphocyte invasion into glioma tissue [17] and because studies have shown successful activation of a cytotoxic T-cell response using dendritic cell-based stimulation by glioma-specific antigens [16]. It is likely that current shortcomings of antiglioma immunotherapy are at least in part due to a failure to adequately address glioma-induced immunosuppression in the local tumor microenvironment [18]. Accordingly, reinvigorated attention has turned to the mechanisms by which glioma cells utilize immune mediators to alter immune behavior. 

We and others have shown that tumor-associated monocytes and microglia (TAMs) are the predominant infiltrating immune cell in malignant glioma and can account for up to 40% of the tumor cell mass [19–21]. Because the frequency of TAMs greatly outnumbers lymphocytes in human gliomas, it is possible that TAMs, under the influence of glioma cells, are playing a major role in the creation of a local tumor microenvironment that is immunosuppressive and promotes glioma growth [22–28]. Considerable efforts to phenotypically and functionally characterize glioma TAMs have led to a delineation between classically activated inflammatory M1-type macrophages with tumoricidal potential from immunosuppressive M2-type macrophages, thought to predominate in the glioma microenvironment [29]. 

**Figure 1:**
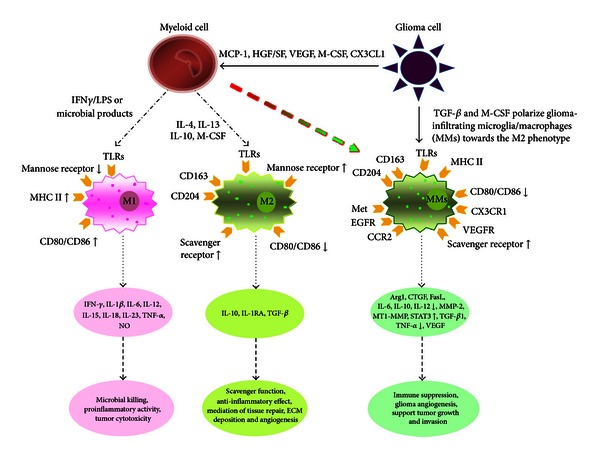
Microglia in glioma are polarized. M1 (classically activated macrophages) and M2 (alternatively activated macrophages) differ with respect to activating signals, receptor expression, cytokine/chemokine production, and biological behavior. When mononuclear/phagocytic cells are stimulated by IFN-*γ* lipopolysaccharides and other microbial products, they differentiate into the M1 phenotype. Microbial products are recognized by pattern recognition receptors (PRRs) on the surface of M1, such as TLRs, and stimulate the production of pro-inflammatory cytokines as well as the expression of receptors that are involved in antigen presentation. When mononuclear/phagocytic cells are activated by IL-4, IL-13, IL-10, and M-CSF, they differentiate into the M2 phenotype. Tumor-derived molecules, such as TGF-*β* and M-CSF, can polarize glioma-infiltrating microglia/microphages (MMs) toward the M2 phenotype and accordingly stimulate the production of anti-inflammatory molecules. Some other glioma-derived molecules, such as MCP-1 and VEGF, can recruit myeloid cells into the tumor site. Published with permission from Li and Graeber [29].

Classically activated M1-type macrophages participate in the coordinated response to immunogenic antigens primarily through production of proinflammatory mediators (such as TNF-*α*, IL-1*Β*, and IL-12), upregulation of cell surface molecules necessary for antigen presentation (including MHC II and costimulatory molecules CD80 and CD86), and an overall enhanced ability to phagocytose pathogenic material [30, 31]. Conversely, alternatively activated M2-type macrophages do not secrete the proinflammatory mediators IL-1*Β* or TNF-*α* [32] and are believed to exert immunomodulation primarily through secretion of the potent immunosuppressive cytokines IL-10, IL-6, and TGF-*β*, downregulation of cell surface molecules necessary for antigen presentation including MHC II, CD80, and CD86, decreased phagocytic capacity, and upregulation of cell surface antigens FasL and B7-H1 both known to stimulate programmed cell death in lymphocytes, among other effects [29, 33, 34]. See Figure 1 for a summary of the M1 and M2 macrophage phenotypes in glioma. 

Recent refinements of this characterization scheme describe a more heterogeneous population of myeloid-derived cells at different stages of maturation, able to suppress multiple phases of the immune response [35]. Termed myeloid-derived suppressor cells (MDSC), cells of this expanded immunosuppressive category have been shown to both perpetuate the glioma-promoting microenvironment as well as distribute peripherally to hinder lymphocyte activation in immune organs [33]. Still, the underlying cellular mechanisms and glioma-TAM interactions dictating the immunomodulatory function of TAMs in glioma remain unclear despite some evidence describing aspects of a complex network of autocrine and paracrine loops of cytokine and chemokine signaling. Is the glioma-promoting relationship [36] between TAMs and the tumor cells present at tumor initiation, or do glioma cells reeducate classically activated TAMs to express an alternative MDSC phenotype at some point in tumor progression? What intrinsic signaling motifs or master regulators of gene expression underlie immunosuppressive TAM phenotypes under the influence of the complex tumor microenvironment? Do certain convergent effector molecules/pathways within TAMs exert disproportionate effects on immunosuppression or glioma facilitation? In this review we will highlight efforts directed at these questions, as their answers may provide information crucial to the development of effective clinical immunotherapy against malignant gliomas. 

## 2. Origins of Tumor-Associated Macrophages in Glioma

Different mononuclear cell-derived populations of distinct lineages exist within the central nervous system (CNS) under pathological conditions. TAMs in human glioma are generally believed to originate from at least two distinct sources. Principal among them are resident microglia, believed to monitor their local neural tissue environment through extensive ramifications, and subsequently to activate a phagocytic phenotype, nearly identical to activated macrophage phenotypes, upon stimulation [37]. A recent fate mapping analysis demonstrated that resident microglia are a distinct lineage that arise from embryonic yolk sac myelomonocytes, which populate the primitive CNS prior to definitive hematopoiesis [38]. Clear evidence has established that activated resident microglia form a large component of macrophages within glioma tissue [19, 39, 40]. A second group of immune cell macrophage precursors in the CNS are peripheral bone marrow-derived mononuclear cells, which colonize the CNS under pathological conditions. Recruitment, engraftment, and subsequent macrophage activation of peripheral mononuclear cells have been established in many experimental models of CNS disease [41–45] and were recently demonstrated to contribute significantly to the macrophage content of human gliomas [19, 46]. Differentiating the lineage origin of individual TAMs isolated from human glioma tissue has proven to be difficult. Most attempts have used FACS sorting of *ex vivo* specimens based on differential levels of CD45 expression in cells coexpressing CD11b, a technique validated in glioma homogenates of chimeric rats [19]. Still, phenotypic and functional differences between these constituent groups of TAMs in gliomas remains largely unknown. 

## 3. Features of Tumor-Associated Macrophages in Glioma

As previously mentioned, the overwhelming predominance of TAMs in the immune infiltrate of both murine and human malignant gliomas has heightened awareness of the influential role these cells may have on both creation of an immunosuppressive tumor microenvironment and facilitation of glioma cell progression [21, 47]. Cumulative research suggests that TAMs within malignant gliomas are dominated by the immunosuppressive M2-type subtype, as the following characteristics have been shown: (1) deficiencies in expected antitumor effector functions of classically activated M1-type macrophages, (2) expression of multiple immunosuppressive antigens and soluble mediators hindering a multifaceted antitumor immune response to glioma tissue, and (3) expression of multiple glioma-promoting mediators including tumor growth and angiogenic factors in addition to stromal remodeling agents, altogether augmenting glioma progression. 

### 3.1. Reduced Antitumor Function in Glioma TAM

**Figure 2:**
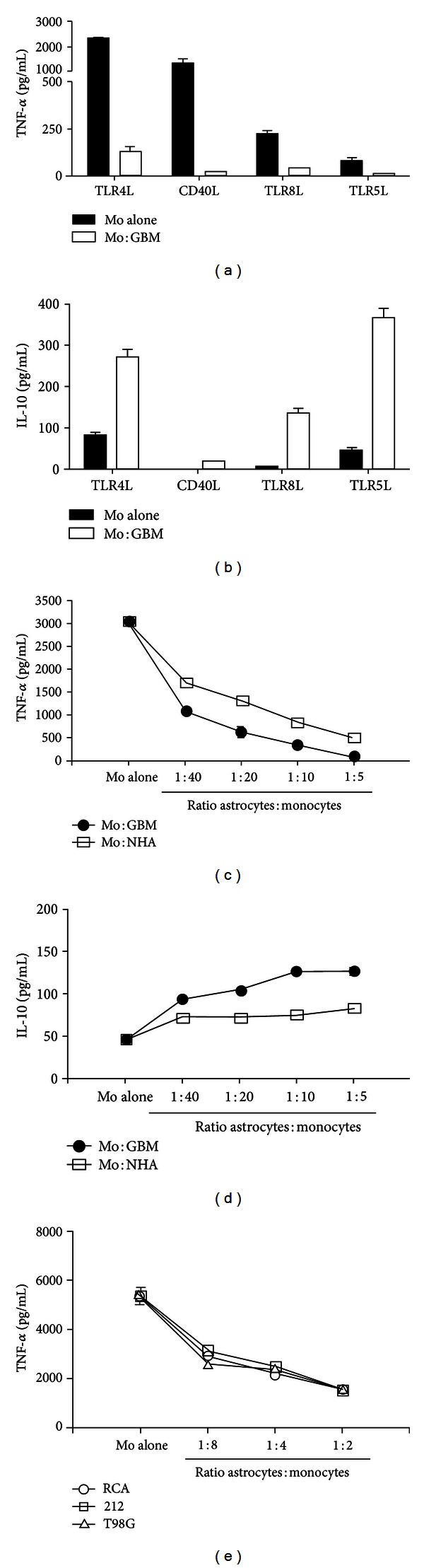
GBM tumor cells suppress monocyte (Mo) activation by a variety of stimuli. ((a) and (b)) *Ex vivo* monocytes were stimulated with the indicated stimuli in the absence or presence of GBM tumor cells, and TNF-*α* and IL-10 secretion was measured after 48 h. Comparable results were seen in five independent experiments. ((c) and (d)) *Ex vivo* monocytes were stimulated with LPS (1 g/mL) in the presence of the indicated ratios of monocytes:GBM tumor cells or NHA. Comparable results were seen in two independent experiments. (e) *Ex vivo* monocytes were stimulated with LPS (1 g/mL) in the presence of the indicated ratios of monocytes and two primary GBM cell lines (RCA and 212) and an extensively passaged GBM cell line (T98G). Comparable results were seen in two independent experiments. SD is represented in all cases. Unstimulated monocytes were used in all assays, and secretion of both TNF-*α* and IL-10 was below the limit of detection. Publised with permission from Kostianovsky et al. [20].

Despite clear evidence of chemotaxis to glioma tumor tissue and subsequent contact with glioma-specific antigens known to be classically immunogenic [18], TAMs in malignant gliomas demonstrate a significant reduction in specific proinflammatory or antitumor effects. Much of this is evidenced by studies showing reductions in secretion of proinflammatory cytokines and increases in secretion of inhibitory cytokines. For instance, our group recently reported that in the presence of malignant glioma cells, there is nearly complete abrogation of TNF-*α* and a significant upregulation of IL-10 secretion by stimulated naïve human monocytes *in vitro* [20]. These findings were subsequently recapitulated *in vivo* when we used our murine glioma model to demonstrate a reduction of TNF-*α* expression by TAMs during late stages of tumor growth [21].  Figure 2 illustrates these findings. 

The mature M1 macrophage marker CD14 serves as a coreceptor of TLR4 and is upregulated in nearly all CNS pathologies [48]. However, downregulation of CD14 has been observed in TAMs in several other cancers, and Rodriguez and Parney et al. demonstrated that monocytes isolated from healthy subjects dramatically reduce expression of CD14 but not CD11b upon exposure to human glioma cell lines [49]. This represents another potential mechanism by which TAMs have diminished antiglioma activity. 

Other deficiencies of glioma TAMs thought to contribute to local immunosuppression are reduction in the expression of HLA and costimulatory molecules. For instance, our group has previously shown significantly reduced CD80 and HLA-DR expression on stimulated naïve human monocytyes when cocultured with GBM cells [20]. This finding is consistent with that of Badie and Schartner who used FACS to demonstrate little to no expression of MHCII, CD80, or CD86 on macrophages freshly isolated from rat gliomas [19]. Furthermore, expression of these costimulatory molecules on TAMs could not be restored by stimulation with IFN-*γ* or LPS [50]. These phenotypic changes on TAMs are likely to be functionally significant *in vivo*, as we have found that monocytes reisolated following coculture with malignant glioma cells demonstrate an inability to activate allogenic CD4^+^ T cells [20]. In addition, there is suppressed secretion of IFN-*γ* from CD4^+^ T cells cultured with GBM-treated monocytes [20]. Similar results regarding downregulation of cell surface molecules and absence of T-cell activation were reported by Rodrigues et al., as well as further demonstrating that, following coculture with malignant glioma cells, human monocytes induce apoptosis in activated autologous T cells [49], a known outcome of incomplete macrophage-T cell-communication.

Glioma TAMs have also been shown to be deficient in phagocytosis. Rodrigues and colleagues demonstrated a significant reduction in the ability to phagocytose bacterial cell wall particles following stimulation in glioma cell-conditioned monocytes, as compared with both astrocyte-conditioned and unconditioned monocytes [49]. In another report, Hussain et al. demonstrated active phagocytosis of opsonized beads in macrophages isolated from *ex vivo *human GBM specimens [32]. This group further attempted to show that TAMs isolated directly from human GBM tumors are deficient of non-MHC-restricted antitumor cellular toxicity through coculture with a target cell line derived from malignant human glioma. Their results indicate minimal cytotoxic ability of these glioma TAMs, as compared to naïve microglia isolated from normal brain tissue [32]. 

### 3.2. TAM-Mediated Immunosuppression in Glioma

In addition to the antitumor effector function deficiencies described earlier, accumulating evidence suggests that glioma TAMs are actively immunosuppressive. Glioma TAMs are now thought to represent an altered phenotype resulting from the directed influence of tumor cells upon immune cells in efforts to produce a favorable tumor microenvironment. Among the immunosuppressive mediators upregulated in glioma TAMs, those that appear to exert a predominate effect include cytokines IL-10, TGF-*β*, and cell surface antigens B7-H1, and FasL [20, 29, 33, 34]. Malignant glioma cells have also been shown to induce tolerance.

**Figure 3:**
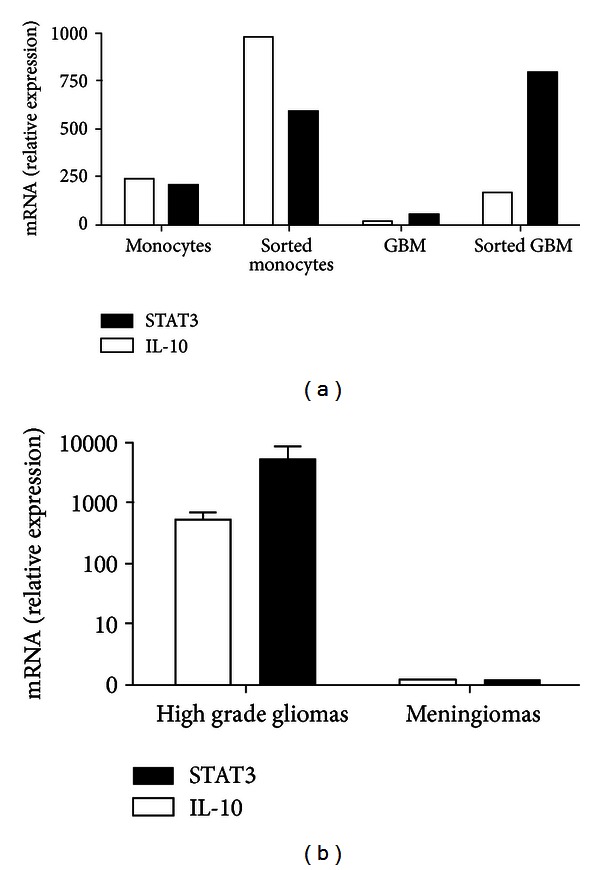
Upregulation of STAT3 and IL-10 in monocytes occurs after coculture with GBM tumor cells *in vitro* and *ex vivo*. (a), *ex vivo* monocytes were stimulated with LPS in the absence or presence of GBM tumor cells for 4 h, at which point monocytes and GBM tumor cells were isolated by FACS. RNA was isolated and levels of IL-10 and STAT3 were measured by quantitative RT-PCR. Similar results were seen in four independent experiments. (b), CD11b^+^CD11c^+^ monocytes/microglia were isolated by FACS from *ex vivo* GBM (*n* = 4) or meningioma (*n* = 2) tumor specimens and RNA was isolated and analyzed for expression of IL-10 and STAT3 by quantitative RT-PCR. Published with permission from Kostianovsky et al. [20].

Our group has previously demonstrated that when cocultured with malignant glioma cells, stimulated naïve human monocytes significantly upregulate expression of IL-10 [20]. Quantitative PCR analysis of these monocytes re-isolated following co-culture demonstrated upregulation of STAT3 as well. Similar results were seen when TAMs from human malignant gliomas isolated directly *ex vivo* were compared with TAMs from meningiomas and these findings are presented in Figure 3. Activation of the transcription factor STAT3 in both glioma cells and glioma TAMs has been suggested to be a key intracellular mediator coordinating the expression of these immunosuppressive molecules [48, 51], leading Brantley and Benveniste to describe STAT3 as a critical “molecular hub” linking multiple pathways in glioma biology [52]. These findings are consistent with earlier work of Wagner et al. who, using multiple molecular techniques, localized both gene expression and IL-10 protein molecules in *ex vivo* GBM tumor specimens overwhelmingly to TAMs, though also present in glioma cells to a much lesser extent [27]. Furthermore, in a recent series of similar experiments, monocytes isolated from healthy subject dramatically increased expression of both IL-10 and TGF-*β* following co-culture with glioma cell lines, as compared to isolated culture as well as co-culture with NHA [49]. Hence, expression of IL-10 by TAMs in glioma tissue appears to be an important immunosuppressive mediator of the glioma microenvironment, and its concomitant expression in glioma cells may serve both as an initial chemotactic agent to recruit monocytes, as well as the driver of feedforward loops of immunosuppressive mediator expression between tumor cells and macrophages within the tumor microenvironment [29, 34]. 

Both glioma cells [53] and TAMs have been shown to express the cell death pathway molecule FasL; indeed Badie et al. demonstrated that nearly every infiltrating monocyte-derived cell in murine glioma models expressed FasL [54]. Furthermore, evidence that apoptotic lymphocytes in the GBM microenvironment express Fas [55] and that microglia induce the apoptotic death of activated T lymphocytes in co-culture [56] together promoted the theory that TAMs may directly exert an immunosuppressive death signal to glioma-infiltrating lymphocytes. This hypothesis is strengthened by the finding that neutralization of FasL results in a significant increase in the number of tumor-infiltrating lymphocytes in a murine glioma model [57]. Other reports, however, have shown that TAMs isolated directly from human glioma resection tissue stained at very low levels or not at all for FasL, leading the authors to conclude that FasL-Fas mediated apoptosis is not a predominant mechanism of immune evasion by TAM in human glioma [32]. Although the precise role of FasL expression on the surface of TAMs within the glioma microenvironment remains unclear, further studies investigating its role in glioma-induced immunosuppression are clearly indicated.

B7-homolog 1 (B7-H1) is a homolog of the costimulatory surface antigens CD80 and CD86 (B7.1 and B7.2) that has been shown to attenuate T-cell receptor function through engagement of the programmed death receptor (PD-1) on the surface of T cells [18]. PD-1 activation on T cells by B7-H1 has been shown to initiate an intracellular signaling cascade resulting in downregulation of T-cell receptor (TCR) signaling [58] and may also promote T-cell apoptosis [63]. Parsa et al. and Wintterle et al. have confirmed near ubiquitous expression of B7-H1 on glioma cells [58, 59], and Rodrigues and colleagues have recently shown B7-H1 expression on human macrophages following co-culture with allogeneic glioma cell lines [49]. Though the precise expression pattern and role of B7-H1 remain unclear, mutual expression of B7-H1 in both glioma and TAM cells may prove a critical mechanism by which lymphocyte suppression is achieved in the tumor microenvironment. 

Although the mechanisms are unknown, there is evidence that malignant gliomas are able to alter monocytes so they become tolerogenic. When human monocytes previously cultured with malignant glioma cells are co-cultured with naïve monocytes, naïve monocytes had a dramatically reduced ability to secrete TNF-*α* in response to stimulation [20]. These findings suggest that inhibition of classically activated antitumor effector functions of glioma TAMs may be a long-lasting regulatory phenotype. 

### 3.3. Nonimmune Glioma Promotion

Glioma cells are known to produce a number of self-supportive factors concurrently with their corresponding cell surface receptors, together acting to promote their own proliferation, migration, angiogenesis, and subsequently tumor extension [60–62]. Indeed, current models of the glioma tumor microenvironment suggest a potent milieu of trophic and immunomodulatory factors bathing all tumor cells and propagating tumor growth through autocrine and paracrine loops of expression and stimulation [29]. Less clear than their lack of effector function or their expression of immunosuppressive mediators, glioma TAMs are increasingly implicated in the contribution of glioma-promoting tumor trophic factors to the local microenvironment. Among the tumor supportive factors potentially secreted by TAMs, TGF-*β*, EGF, and HGF/SF have drawn the most attention, though dissecting the precise role of TAMs in the production of these trophic factors remains to be accomplished. 

Tissue growth factor beta (TGF-*β*) is an extremely potent immunosuppressive and transformative cytokine whose expression is mostly associated with glioma cells themselves [63, 64] and is believed to have a major influence in directing the alternatively activated immunosuppressive phenotype of TAMs [65]. In addition to the well-documented immunomodulatory and tumor cell tropic effects of TGF-*β* through induction of VEGF and FGFs [66], TGF-*β* may also promote tumor cell migration and invasion through induction of MMP expression in conjunction with suppression of tissue inhibitor of metalloproatease expression [67], together affecting stromal remodeling to facilitate invasion. Microglia have been shown to produce TGF-*β* isoform 1 (TGF-*β*1) under certain pathological conditions including neuritis and trauma [68, 69]. Using *in situ* hybridization, Kiefer et al. localized the expression of the TGF-*β*1 isoform to activated glioma TAMs in a murine model, suggesting to the authors this isoform's involvement in a mutually reinforcing paracrine loop with glioma cells [70]. Building upon this hypothesis, Li and Graeber proposed that, whereas glioma-derived TGF-*β* exerts immunosuppression by driving alternative polarization in TAMs, TGF-*β* produced by the glioma TAMs may promote tumor growth and invasion by stimulating the upregulation of its own cognate receptors TBRI and TBRII on glioma cells [29] enabling a more potent trophic response to the high concentration of TGF-*β* proposed to exist in the glioma microenvironment.

Epidermal growth factor expression and stimulation of its cognate receptor (EGF/EGFR) have emerged as a pivotal signaling mechanism in high grade glioma. EGFR amplification is seen in approximately 50% of GBM, and in approximately 50% of those tumors the glioma cells express EGFRvIII, a mutant receptor that persistently activates downstream immunosuppressive pathways including those involving STAT3 [71]. In two separate efforts, activated microglia from a murine glioma model demonstrated expression of EGFR [72] as well as low levels of EGF secretion [73]. These initial findings again position TAMs within a potential paracrine network with glioma cells, acting to reinforce expression of both EGF and EGFR on glioma cells to promote tumor progression. 

Hepatocyte growth factor/scatter factor acts exclusively through the tyrosine kinase receptor c-Met and expression of both the soluble ligand and receptor has been demonstrated in both *ex vivo* human glioma and TAM cells [74, 75]. Kunkel et al. used combined *in situ* hybridization with fluorescence immunohistochemistry to demonstrate expression of both HGF/SF and c-Met in a majority of TAMs isolated from human *ex vivo *GBM specimens [75]. Badie et al. demonstrated *in vitro* that glioma-derived HGF/SF is a potent chemotactic agent on microglia [61] postulating that tumor-secreted HGF/SF acting upon TAM c-Met receptors may be a major mechanism by which glioma tissue recruits monocytes to commandeer toward the construction of a favorable microenvironment. Stimulation of c-Met by HGF/SF in human GBM cell lines has been shown to increase proliferation and invasive motility [74] and furthermore to induce angiogenesis in murine glioma tissues [76], yet it remains unclear if this latter effect is mediated through direct action on glioma endothelial cells or through induction of VEGF. Indeed, in separate efforts, radiation and hypoxia were shown to induce c-Met expression in glioma cells, further supporting its role in glioma tumor angiogenesis [77, 78]. Altogether these findings again suggest a mutually reinforcing network of HSF/SF upon c-Met paracrine signaling between glioma cells and TAMs, whereby glioma cells recruit monocytes in the alternatively activating tumor microenvironment to subsequently derive trophic stimulation by alternatively mature TAM secretion of HGF/SF. 

## 4. Dynamics of Glioma-Tam Interaction

**Figure 4:**
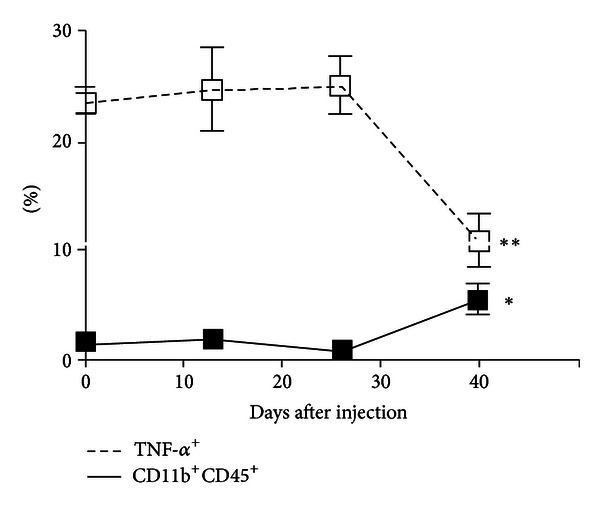
Frequency and functional changes in tumor-associated macrophages and microglia (TAMs) in *ex vivo* tumor specimens over the course of tumor development. While the percentage of TAMs (CD11b^+^CD45^+^ cells) was increased by the final time point (1.1% versus 5.6%, *P* = 0.017), their functional capacity was impaired as measured by TNF-*α* expression (25.2% versus 10.9%, *P* = 0.007). Thirteen mice were analyzed at 13 dpi (*n* = 4), 26 dpi (*n* = 4), and at 29 dpi upon exhibiting clinical tumor morbidity (*n* = 1) or at 40 dpi (*n* = 4). Differences between the means at each time point were tested using two-sided *t*-tests with unequal variances. *P < 0.05, **P < 0.01, ***P < 0.001. Published with permission from Kennedy et al. [21].

A major shortcoming of the efforts to understand glioma-associated macrophages remains a paucity of data describing the dynamics of glioma cell and macrophage interactions. Most of the findings reviewed to this point are based upon *ex vivo* human samples taken at the time of surgical resection, or similarly upon murine specimens harvested at a late-stage time point when glioma tumor mass is grossly apparent. Both of these scenarios likely represent end-stage tumors wherein the tumor has advanced to aggressive behavior, abetted by immunosuppressive glioma-promoting TAMs, as this review has demonstrated. Little data is available to suggest if the partnership between glioma cells and alternatively activated M2 TAMs arises at tumor onset and therefore contributes early in tumor formation, or if TAMs initially manifest a classically activated M1 phenotype to combat tumor development until some critical point when tumor-derived mediators overwhelmingly direct M2 polarization, followed by rapid tumor progression and clinical presentation. Recent efforts by our group have sought to address these questions through kinetic studies of the infiltration and function of immune cells in a murine glioma model, both within the tumor microenvironment and peripherally [21]. Using multiparameter FACS, we assessed TAM frequencies at early, intermediate, and late time points following injection of a PDGF-expressing retrovirus (13, 26, and 40 days after injection) and demonstrated little change in TAM frequency between early and intermediate time points, despite a substantial increase in TAM frequency in the final time point. Furthermore, at each time point, TAM function status was assessed through evaluation of TNF-*α* secretion. Of great interest, no change was observed in the proportion of TAMs secreting TNF-*α* between the early and intermediate time points, though a 2.5-fold reduction in the TNF-*α* -secreting TAMs was evident by the final time point. These findings are summarized in Figure 4. Taken together in the context of findings referenced in this review, these observations may suggest a fulcrum in TAM activation status at some point between the intermediate and late time points, whereafter glioma cells are able to tip the balance toward an alternatively activated M2 TAM phenotype, thereby amplifying autocrine and paracrine loops successful in recruiting greater numbers of TAMs which do not express TNF-*α*. 

Peripheral immune changes were investigated with splenic preparations, evaluated for presence of IFN-*γ*—producing CD4^+^ T cells and regulatory T cells (CD4^+^FoxP3^+^). Between the early and intermediate time points, the proportion of IFN-*γ*—producing CD4^+^ T cells, increased modestly and sustained this elevated frequency through the late time point. Interestingly, the proportion of regulatory T cells increased significantly between the early and intermediate time points and also remained elevated, though slightly decreased from peak frequency, through the late time point. Moreover, the proportion of these T-reg cells that expressed IL-10 mirror the overall frequency, increasing significantly between early and intermediate time points and exhibiting a sustained effect through the late time point. These findings suggest that peripheral immunosuppressive changes may precede those in the tumor microenvironment. Efforts of our group and others have established regulatory T cells and others as major immunosuppressive cellular mediators implicated both locally and systemically in glioma patients [79–81]. The crosstalk between glioma-associated TAMs and peripheral or glioma-infiltrating Tregs is likely complex, and our work may suggest that early Tregs may have a role in the induction of the later M2 TAM phenotype. Our group and others are continuing to pursue further characterization of these intercellular relationships, leading us toward a more nuanced understanding of the process of creating the immunosuppressive glioma microenvironment.

## 5. Underlying Mechanisms of Alternatively Activated TAMs

As this review suggests, the creation of a potent immunosuppressive, glioma conducive, tumor microenvironment at least in part results from the complex interplay of glioma cells and alternatively activated TAMs that involve many immune mediators with pleiotropic affects. While extensive efforts to characterize glioma TAMs have established many of the individual immunosuppressive and tumor permissive features specific to these cells, an understanding of the molecular pathways and signaling molecules affecting gene expression which lead to the altered phenotype of glioma TAMs is not known. Intracellular mediators at points of signaling convergence within glioma TAMs and the molecular products which result from their activation present potential targets for immunotherapy-based strategies directed against malignant gliomas. Furthermore, recent evidence highlighting prognostic differences among distinct molecular subtypes of glioma, including those with 1p/19q codeletion, isocitrate dehydrogenase (IDH) mutations, and differential MGMT methylation status [82], raises the possibility of subtype specific differences in both glioma cell and TAM expression profiles, and therefore in the composition of the tumor microenvironment; such discrepancies remain to be explored. 

At this point, recognition of differential activation of the signal transducer and activator of transcription 3 protein (STAT3) within both glioma cells [83] and glioma TAMs [84] is the most compelling evidence of a single gene involved with multiple immunosuppressive signaling pathways in glioma induced immunosuppression. STAT3 activation in glioma TAMs is induced by many mediators known to compose the local tumor microenvironment such as IL-10, IL-6, EGF, and FGF [85]. Activated STAT3 is known to reduce the expression of surface molecules necessary for antigen presentation such as MHCII, CD80, and CD86 [86], as well as to increase the expression of many M2 specific immunomodulatory mediators including IL-10, EGF, VEGF, and various MMPs [51, 52]. Therefore, STAT3 may serve as a critical “molecular hub” [52] linking multiple pathways unique to alternatively activated M2 type TAMs. Furthermore, STAT3 target molecules such as IL-10 and IL-6 have been shown to activate STAT3 [51], leading Li and Graeber to propose a feed-forward mechanism which may account for the constitutive activation of STAT3 in both glioma cells and glioma-infiltrating TAMs [29]. 

Though STAT3 activation appears to play a key role in generating and perpetuating the M2-type TAMs in gliomas, it is unclear whether a single dominant molecule or a complex network of molecules is responsible for the immunosuppressive phenotype of glioma TAMs. Our group therefore conducted a microarray-based approach of sorted human monocytes after co-culturing with malignant gliomas in an attempt to better characterize these pathways. Through extensive pathway analyses and network exploration, we identified a small subset of novel candidate genes that could be responsible for glioma-induced dysfunction of TAMs. To demonstrate that these candidate genes identified by the microarray analysis were important *in vivo*, we then isolated TAMs from patients with primary and recurrent malignant gliomas and confirmed their differential expression patterns *ex vivo*. Our next step is to use siRNA to block these candidate genes in our GBM: monocyte co-culture model to determine if monocyte function can be restored. Importantly, preliminary analysis confirms that STAT3 is not among our candidate genes, suggesting that multiple pathways contribute to TAM dysfunction in gliomas. We hope that the identification of genes differentially expressed in glioma TAMs may give us a better understanding of the pathways driving M2-type polarization and lead to new, more effective targets for glioma based immunotherapy.

## 6. Conclusions 

Are tumor-associated monocytes/microglia in malignant gliomas friends or foes? Although a comprehensive answer to this question remains elusive, the considerable efforts described in this review seem to cast TAMs in glioma as a formidable foe, espousing an altered activation state within the local tumor microenvironment characterized by deficiencies in antitumor effector functions, upregulation of potent immunosuppressive mediators, and participation in tumorigenic loops of paracrine signaling involving expression of trophic factors and their cognate receptors. Although the dynamics of this malign partnership between glioma cells and TAMs remain unclear over the course of tumor progression, a turning point seems to occur late in tumor development, perhaps providing a better opportunity for clinically based immunotherapy. Given the compelling evidence that TAMs contribute significantly to the creation and maintenance of immunosuppression and tumor progression, it is unlikely that clinically effective immunotherapy against malignant gliomas will be achieved until we gain a better understanding of how to influence TAM function in the local tumor microenvironment.
